# Bioactive Pentacyclic Triterpenes from the Stems of *Combretum laxum*

**DOI:** 10.3390/molecules13112717

**Published:** 2008-11-01

**Authors:** Eder Bisoli, Walmir Silva Garcez, Lidilhone Hamerski, Caroline Tieppo, Fernanda Rodrigues Garcez

**Affiliations:** Departamento de Química, Universidade Federal de Mato Grosso do Sul, Campo Grande, MS, Brasil, 79070-900; E-mails: ederbisoli@yahoo.com.br (E. B.), wgarcez@nin.ufms.br (W-S. G.), lidihamerski@ig.com.br (L. H.), carol_tieppo@hotmail.com (C T.)

**Keywords:** *Combretum laxum*, Combretaceae, triterpenes, antifungal activity

## Abstract

Two new triterpene glucosides, β-d-glucopyranosyl 2α,3β,24-trihydroxyolean-12-en-28-oate and β-d-glucopyranosyl 2α,3β,23,24-tetrahydroxyurs-12-en-28-oate, in addition to nine known compounds belonging to three different triterpene classes (oleanane-, ursane- and lupane-type) have been isolated from the stems of a specimen of *Combretum laxum* growing in the “Pantanal” of the central-western region of Brazil. Among the known triterpenes, β-d-glucopyranosyl 2α,3β,6β-trihydroxyolean-12-en-28-oate is reported for the first time in the Combretaceae, while bellericoside and asiatic acid are described for the first time in the genus *Combretum*. The structures of the isolated compounds have been established on the basis of spectral techniques (1D-, 2D-NMR and MS). Their *in vitro* antifungal activities against standard strains of *Candida albicans*, *C. krusei* and *Cryptococcus neoformans* were also evaluated in this work.

## Introduction

The state of Mato Grosso do Sul, located in the central-western region of Brazil, is home of three major biomes, namely “Cerrado”, “Pantanal” and tropical forest. The “Pantanal”, the world’s largest wetland area, with a vast range of biodiversity, is found in Brazil in only two states: Mato Grosso and Mato Grosso do Sul, the latter comprising 2/3 of the total area of the Brazilian “Pantanal”, which is 150,355 km^2^ [1]. As part of our ongoing program to assess the chemical diversity of plants of the family Combretaceae which are distributed in the first two ecosystems [2,3,4], we have investigated the stems of *Combretum laxum*, a climbing shrub occurring in the “Pantanal” region and popularly known as “pombeiro-branco”. Amongst the 18 genera comprising the Combretaceae, the genus *Combretum* is the largest, with 250 species, which are mostly native to tropical and subtropical regions [5]. Plants of this genus are known as a rich source of secondary metabolites, in particular triterpenes and their glycosides, phenanthrenes, bibenzyls, stilbenes, flavonoids, in addition to other aromatic compounds, some of which display anticancer, antimicrobial and hepatoprotective activities, among others [6,7,8,9,10,11,12,13,14,15]. To date, only one chemical constituent, *trans*-4-hydroxyproline betaine, was reported to occur in a Venezuelan specimen of *C. laxum* [16]. Herein we describe the isolation and characterization of two new triterpene glucosides, β-d-glucopyranosyl 2α,3β,24-trihydroxyolean-12-en-28-oate and β-d-glucopyranosyl 2α,3β,23,24-tetrahydroxyurs-12-en-28-oate from the title plant, along with nine known oleanane-, ursane- and lupane-type triterpenoids, and the evaluation of their antifungal activities.

## Results and Discussion

Compound **1** was obtained as a white, amorphous solid, and displayed a quasi-molecular ion at *m/z* 685.3755 [M+Cl]^-^ in the HRESIMS, consistent with the molecular formula C_36_H_58_O_10_ (calcd. for C_36_H_58_O_10_-Cl 685.37204). The ^1^H-NMR spectrum of **1** showed six aliphatic methyl singlets at δ 0.85, 0.87, 0.97, 1.06, 1.18 and 1.54 and a broad singlet at δ 5.38 assignable to an olefinic hydrogen. The ^13^C and DEPT NMR spectra exhibited signals for six methyls, eleven methylenes (two oxymethylenes), eleven methines (one olefinic, seven oxymethines) and eight quaternary carbons (one olefinic and one ester carbonyl carbon), of which six were attributed to a sugar unit and 30 were consistent with an triterpene aglycon moiety. The presence of a Δ^12^-double bond, already inferred from the broad singlet at δ 5.38 ppm in the ^1^H-NMR spectrum was corroborated by resonances at 122.7 and 144.1 in the ^13^C-NMR spectrum, assigned to C-12 and C-13, respectively, of an olean-12-ene-type skeleton [17]. The presence of hydroxyl substituents at C-2 and C-3 was evident from both the chemical shifts and *J* values of the hydrogens ascribable to H-2 (δ 4.33, m) and H-3 (δ 3.54, d, *J* = 9.3 Hz), which showed connectivities in the HSQC spectrum with the oxymethine carbons at δ 68.6 and δ 85.7, respectively. The large coupling constant (9.3 Hz) between H-2 and H-3 pointed to their axial disposition, thereby indicating that the 2- and 3- hydroxyl groups were both equatorially (2α, 3β) oriented. Furthermore, the ^1^H-NMR spectrum also revealed two one-proton doublets [δ 3.69 and δ 4.42 (*J* = 11.0 Hz)] assignable to diastereotopic hydroxymethylene hydrogens, which in turn showed cross-peak correlations with a carbon signal at δ 65.7 in the HSQC spectrum. This information, along with an upfield shifted methyl carbon signal at δ 24.1 ascribable to C-23 and a downfield quaternary carbon resonance at δ 44.0 attributable to C-4 strongly suggested the location of this third hydroxyl substituent at C-24 [17]. This assumption could be confirmed by connectivities observed in the HMBC spectrum between this carbon signal and H-3 (δ 3.54) and methyl-23 (δ 1.54) resonances. The ^13^C-NMR data also allowed the assignment of the pyranose form of D-glucose [18], while the magnitude of the coupling constant of the ^1^H-NMR doublet at δ 6.29 (*J* = 7.9 Hz) indicated an α-orientation of the anomeric hydrogen of the glucosyl residue, thus establishing a β-configuration for the glucopyranosyl unity. The chemical shift value of its anomeric carbon (δ 95.8) supported the fact that compound **1** was an ester glucoside, since the anomeric carbons of *O*-glucosides are observed around 100 ppm, independently of the nature of the sugar residue [19]. This information, along with the ester carbonyl carbon resonance (δ 176.4) and the remaining carbon resonances of the triterpene skeleton indicated the likely location of the sugar attachment at C-28. The aforementioned data could be satisfactorily assembled to establish that **1** was the C-28 glucopyranosyl ester derivative of 2α,3β,24-trihydroxyolean-12-en-28-oic acid, whose aglycon, known as hyptatic acid-A, was previously isolated from *Hyptis capitata* (Labiatae) [17] and also obtained from chemical transformation of maslinic acid [20]. Indeed, the spectral data of **1** closely resembled those of the corresponding oleanane triterpene acid [17,20], except for the absence in the latter of the signals attributed to the glucosyl moiety in **1**. The replacement of the signal for the C-28 carboxyl observed in the triterpene aglycon δ 181.8 [20] by an upfield shifted ester carbonyl carbon at δ 176.4, along with the long-range coupling discernible in the HMBC spectrum between this carbon and the anomeric hydrogen of the sugar (δ 6.29) further supported the linkage site of the glucopyranosyl moiety at C-28. Thus, the structure of **1** was determined to be β-d-glucopyranosyl 2α,3β,24-trihydroxyolean-12-en-28-oate.

The structures of the five additional oleanane-type triterpenes isolated from *Combretum laxum* were established on the basis of spectral analyses and by comparison with previously reported data or authentic samples as arjunolic acid (**2**) [3,4,17], arjunglucoside II (β-d-glucopyranosyl 2α,3β,23-tri- hydroxyolean-12-en-28-oate, **3**) [4,21], bellericoside (β-d-glucopyranosyl 2α,3β,23,24-tetrahydroxy-olean-12-en-28-oate, **4**) [22], chebuloside II (β-d-glucopyranosyl 2α,3β,6β,23-tetrahydroxyolean-12-en-28-oate, **5**) [23] and β-d-glucopyranosyl 2α,3β,6β-trihydroxyolean-12-en-28-oate (**6**) [24]. Compound **6**, which had previously only been isolated from *Siphoneugena densiflora* (Myrtaceae) [24], is therefore described for the first time in the Combretaceae. Also worthy of mention is the fact that although **4** has been shown to occur in other members of the Combretaceae, e.g. in *Terminalia* bellerica [22], it was hitherto unreported in the genus *Combretum*, while chebuloside II and arjunolic acid have been described in only one (*C. quadrangulare*) [12] and two species (*C. quadrangulare* and *C. leprosum*) [6,11] of this genus, respectively.

The structures of compounds **7** and **8** were readily discernible from both the ^1^H- and ^13^C-NMR spectra as those of the known ursane-type triterpenoids asiatic acid and β-d-glucopyranosyl 2α,3β,23-trihydroxy-urs-12-en-28-oate (quadranoside IV), respectively, whose spectral data were in accordance with those of an authentic sample [3] and those published in the literature [13]. In spite of the presence of asiatic acid either in members of the Combretaceae (e.g., in the genus *Terminalia* [3]) or in other plant families, this constitutes the first report of its isolation in the genus *Combretum*, whereas this is the second reported occurrence of quadranoside IV in this genus, where it was first described in *C. quadrangulare* [13]. Compound **9** was obtained as the minor component of an unresolved mixture with bellericoside (**4**). The comparison of the ^13^C-NMR spectrum of this mixture to that of pure compound **4** enabled assignment of its carbon resonances. The remaining ones were indicative that **9** was the urs-12-ene derivative analogue of bellericoside. This assumption was revealed by the typical resonance values of C-12 and C-13 of an ursane-type triterpene observed at δ 125.9 and 138.4, respectively [17] and the characteristic signals attributable to the ring E carbons, namely δ 53.2 (C-18), δ 39.3 (C-19), δ 39.1 (C-20), δ 30.7 (C-21) and δ 36.7 (C-22), as well as methyls 29 (δ 17.5) and 30 (δ 21.2) [17], which in turn were very similar to the corresponding signals observed in the spectrum of quadranoside IV (**8**). Resonance values of the remaining carbons in the structure of **9** were essentially the same as those of bellericoside (**4**). The foregoing account therefore allowed assignment of the structure of **9** as β-d-glucopyranosyl 2α,3β,23,24-tetrahydroxyurs-12-en-28-oate. This triterpene glucoside was previously described as being part of the structure of the castanopsinins A-H, galloyl and hexa-hydroxydiphenoyl-type ellagitannins possessing a mixture of olean-12–ene and ursan-12-ene triterpene glucoside cores in their molecules, which were isolated from *Castanopsis cuspidate* var. *sieboldii* (Fagaceae) [25].

**Figure 1:**
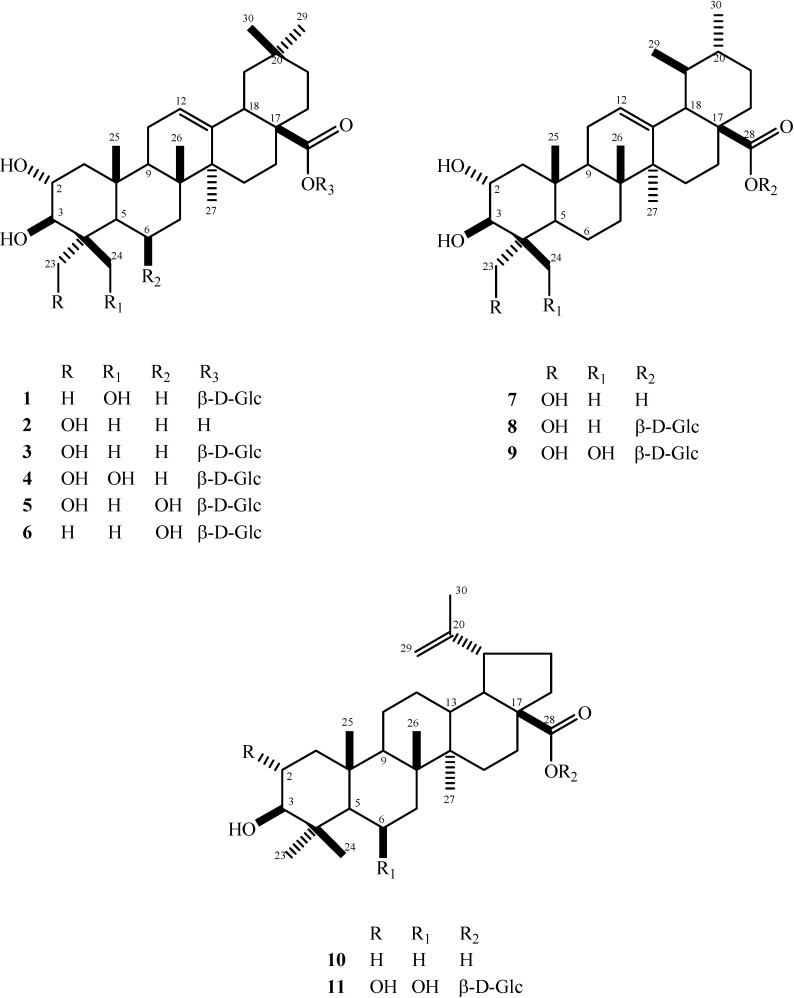
Structures of the triterpenoids **1** – **11** isolated from *C. laxum.*

The lupane-type triterpenes **10** and **11** obtained in the present work were found to be identical in their spectral properties with betulinic acid (data compared with those of an authentic sample) [3,4] and β-d-glucopyranosyl 2α,3β,6β-trihydroxy-lup-20(29)-en-28-oate (quadranoside I) [13], respectively. The latter has only been found in *C. quadrangulare* and *Vochysia pacifica* (Vochisiaceae) [26] and in spite of the wide distribution of betulinic acid in other plant genera, its previous occurrence in the genus *Combretum* has only been reported in *C. quadrangulare* [14]. The structures of all the isolated compounds are summarized in Figure 1. 

Following isolation, compounds **1**, **2**, **5**, **6**, **10**, **11** and the mixtures of **2** and **7**, **3** and **8**, **4** and **9** were further evaluated for their antifungal properties *in vitro* against *Candida krusei*, *C. albicans* and *Cryptococcus neoformans*, using the microbroth dilution assay. All samples displayed antifungal activities against the strains tested and their minimal inhibitory concentration (MIC) values are given in Table 1. Arjunolic acid (**2**) was the most active triterpenoid assayed, with MIC values = 50 μg/mL against the three fungal strains, while betulinic acid (**10**) showed strongest activities against *Candida albicans* and *Cryptococcus neoformans* (MIC = 100 μg/mL) and chebuloside II (**5**) against *C. neoformans* (MIC = 100 μg/mL). The crude ethanol extract, on the other hand, was shown to be inactive against these strains. Although crude extracts from a number of plants of the family Combretaceae, mostly occurring in the African continent, have shown antifungal properties against yeasts and/or filamentous fungi [27,28,29,30,31], only a few works have reported on the antifungal activities of pentacyclic triterpenes obtained from these species, most of them belonging to the genus *Terminalia* [32,33,34]. Recently, in an antifungal bioassay-guided fractionation of crude extracts of the leaves of *Combretum nelsonii*, a mixture of arjunolic and asiatic acids was reported to show strong activities against *Candida albicans* and *Cryptococcus neoformans*, being also active against filamentous fungi [35]. However, these triterpenes (**2** and a mixture of **2** and **7**) showed weak activities against the strains of *Candida albicans* and *Crytococcus neoformans* used in the present work. The results presented herein add new biological activity data of the secondary metabolites obtained from members of the genus *Combretum.*

**Table 1:** Minimal inhibitory concentration (MIC) values (μg/mL) of triterpenes isolated from *C. laxum* against *Candida* and *Cryptococcus* strains using the microbroth dilution assay.

Compounds	*Candida albicans*	*Candida krusei*	*Cryptococcus neoformans*	
	ATCC 90028	ATCC 6258	ATCC 32045	
**1**	200	200	200
**2**	50	50	50
**5**	200	200	100
**6**	200	200	200
**10**	100	200	100
**11**	200	200	200
**2/7**	200	200	200
**3/8**	200	200	200
**4/9**	200	200	200
amphotericin B	0.25	0.50	0.50

## Experimental

### General

Optical rotations were determined on a Perkin Elmer 341 polarimeter (Na filter, λ = 589 nm). ^1^H- and ^13^C- 1D and 2D NMR spectra were recorded in pyridine-*d_5_* at 300 MHz (^1^H) and 75 MHz (^13^C) on a Bruker DPX-300 spectrometer. Standard pulse sequences were used for homo- and heteronuclear correlation experiments. HRESIMS spectrum was acquired in negative ion mode on an UltrOTOF-Q instrument (Bruker Daltonics). Column chromatography procedures were performed on silica gel 70-230 mesh and 230-400 mesh, RP-18 silica gel 230-400 mesh and Sephadex LH-20. Reversed phase semi-preparative HPLC separations were performed with a Shimadzu LC-6AD pump, using a RP-18, 21.6 x 250 mm, 5 μm particle size, Phenomenex Luna column, with a flow rate of 10 mL or 14 mL min^-1^ and monitoring at 210 nm.

### Plant Material

Stems of *Combretum laxum* Jacq. were collected in Miranda (“Pantanal” of Mato Grosso do Sul), Brazil, in October 2004. The plant material was identified by MSc. Ubirazilda M. Resende, CGMS Herbarium, Universidade Federal de Mato Grosso do Sul, Brazil, where a voucher specimen (No. 18102) is deposited.

### Extraction and Isolation

Air-dried and powdered stems of *C. laxum* (4,375 g) were extracted at room temperature with EtOH. After concentration *in vacuo*, the residue obtained from the EtOH extract was subsequently partitioned between MeOH/H_2_O 9:1 and hexane; MeOH/H_2_O 1:1 and CH_2_Cl_2_; MeOH/H_2_O 1:1 and EtOAc and MeOH/H_2_O 1:1 and *n*-BuOH to give the corresponding hexane, CH_2_Cl_2_, EtOAc and *n*-BuOH phases. Betulinic acid (**10**, 200.0 mg) was obtained from the CH_2_Cl_2_ phase (9.2 g), after a series of column chromatography procedures over silica gel (70-230 mesh) eluted with hexane, CH_2_Cl_2_, EtOAc and EtOAc/MeOH 20%. The EtOAc phase (7.86 g) was chromatographed on a silica gel (70-230 mesh) column eluted with hexane, CH_2_Cl_2_, EtOAc and EtOAc/MeOH 20%. Twenty-nine fractions of 250 mL each were collected and those exhibiting similar TLC profiles were combined to give, altogether, eight fractions (A-H). Fraction D (EtOAc, 1.31 g) was subjected to column chromatography on silica gel (230-400 mesh) eluted with CHCl_3_/MeOH 10% to yield twenty-eight fractions of 20 mL each. The second fraction (24.6 mg) consisted of a mixture of the isomeric triterpene acids **2** (major compound) and **7** and the sixth one (409.6 mg) was further separated on Sephadex LH-20 (MeOH) followed by semi-preparative HPLC (CH_3_CN/H_2_O 38:62) to afford **1** (16.3 mg), **6** (10.3 mg), **11** (8.0 mg) and a mixture of the isomeric triterpenes **3** (major compound) and **8** (25.0 mg). The *n*-BuOH phase (25.5 g) was subjected to column chromatography on RP-18 silica gel eluted with MeOH/H_2_O (6:4 to pure MeOH), to furnish seven fractions of 500 mL each. The fourth fraction (MeOH/H_2_O 8:2, 0.5 g) was rechromatographed on a Sephadex LH-20 column (MeOH) and further purified by semi-preparative HPLC (CH_3_CN/H_2_O 30:70) to yield **4** (5.8 mg), **5** (19.0 mg), additional amounts of a mixture of **3** and **8** (24.2 mg) and a mixture of **4** (major compound) and **9** (4.8 mg) which proved difficult to be separated in its individual components on subsequent chromatographic procedures.

β-*d-Glucopyranosyl 2α,3β,24-trihydroxyolean-12-en-28-oate* (**1**): colorless amorphous powder; 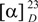: + 22.79^o^ (*c* 0.17, MeOH); HRESIMS *m/z* 685.3755 [M+Cl]^-^ (calcd. for C_36_H_58_O_10_Cl 685.3720); ^1^H-NMR: δ 4.33 (1H, m, H-2); 3.54 (1H, d, *J* = 9.3 Hz, H-3); 5.38 (1H, brs, H-12); 3.16 (1H, dd, *J* = 14.3, 3.8 Hz, H-18); 3.69 (1H, d, *J* = 11.0 Hz, H-24a); 4.42 (1H, d, *J* = 11.0 Hz, H-24b); 1.54 (3H, s, H-23); 0.97 (3H, s, H-25); 1.06 (3H, s, H-26); 1.18 (3H, s, H-27); 0.87 (3H, s, H-29); 0.85 (3H, s, H-30); sugar signals: δ 6.29 (1H, d, *J* = 7.9 Hz, H-1’); 4.00 (1H, m, H-5’); 4.18-4.45* (5H, m H-2’, H-3’, H-4’, H-6’) *overlapping signals; ^13^C-NMR: δ 47.8 (C-1); 68.6 (C-2); 85.7 (C-3); 44.0 (C-4); 56.5 (C-5); 19.3 (C-6); 33.4 (C-7); 40.0 (C-8); 48.3 (C-9); 38.3 (C-10); 24.1 (C-11); 122.7 (C-12); 144.1 (C-13); 42.1 (C-14); 28.2 (C-15); 23.4 (C-16); 47.0 (C-17); 41.7 (C-18); 46.2 (C-19); 30.7 (C-20); 34.0 (C-21); 32.5 (C-22); 24.1 (C-23); 65.7 (C-24); 17.3 (C-25); 17.4 (C-26); 26.0 (C-27); 176.4 (C-28); 33.1 (C-29); 23.6 (C-30); sugar signals: δ 95.8 (C-1’); 74.1 (C-2’); 78.9 (C-3’); 71.1 (C-4’); 79.3 (C-5’); 62.3 (C-6’).

*Arjunolic acid* (**2**) and *Asiatic acid* (**7**) as a mixture: colorless amorphous powder; ^1^H- and ^13^C-NMR data in accordance with those of an authentic sample and with the literature [3,4,17]. Compound **2**: ^13^C-NMR: δ 47.6 (C-1); 68.8 (C-2); 78.2 (C-3); 43.6 (C-4); 47.9 (C-5); 18.5 (C-6); 32.8 (C-7); 39.8 (C-8); 48.1 (C-9); 38.4 (C-10); 23.6 (C-11); 122.4 (C-12); 144.8 (C-13); 42.2 (C-14); 28.2 (C-15); 23.9 (C-16); 46.6 (C-17); 41.9 (C-18); 46.3 (C-19); 30.9 (C-20); 34.1 (C-21); 33.2 (C-22); 66.4 (C-23); 14.3 (C-24); 17.3 (C-25); 17.5 (C-26); 26.1 (C-27); 180.2 (C-28); 33.2 (C-29); 23.7 (C-30). Compound **7**: ^13^C-NMR: δ 47.6 (C-1); 68.8 (C-2); 78.2 (C-3); 43.6 (C-4); 48.2 (C-5); 18.5 (C-6); 32.8 (C-7); 40.0 (C-8); 47.6 (C-9); 38.3 (C-10); 23.6 (C-11); 125.5 (C-12); 139.2 (C-13); 42.2 (C-14); 28.6 (C-15); 24.8 (C-16); 48.0 (C-17); 53.5 (C-18); 39.4 (C-19); 39.3 (C-20); 31.0 (C-21); 37.4 (C-22); 66.4 (C-23); 14.3 (C-24); 17.4* (C-25); 17.4* (C-26); 23.8 (C-27); 180.6 (C-28); 17.3* (C-29); 21.3 (C-30) *interchangeable signals.

*Arjunglucoside II* (**3**) and *Quadranoside IV* (**8**) as a mixture: colorless amorphous powder; ^1^H- and ^13^C-NMR data in accordance with those of an authentic sample and/or with the literature [4,13,21]. Compound **3**: ^1^H-NMR: δ 4.25* (1H, m, H-2); 4.28* (1H, m, H-3); 5.43 (1H, brs, H-12); 3.19 (1H, dd, *J* = 13.5, 4.2 Hz, H-18); 3.72 (1H, d, *J* = 10.6, H-23a); 4.22* (1H, d, *J* = 10.6, H-23b); 1.07 (3H, s, H-24); 1.10 (3H, s, H-25); 1.16 (6H, s, H-26 e H-27); 0.90 (3H, s, H-29); 0.88 (3H, s, H-30); sugar signals: δ 6.33 (1H, d, *J* = 9.0 Hz, H-1’); 4.21 (1H, m, H-5’); 4.22-4.46* (5H, m H-2’, H-3’, H-4’, H-6’) *overlapping signals; ^13^C-NMR: δ 47.6 (C-1); 68.8 (C-2); 78.1 (C-3); 43.5 (C-4); 48.0 (C-5); 18.4 (C-6); 32.7 (C-7); 39.9 (C-8); 47.7 (C-9); 38.3 (C-10); 23.8 (C-11); 122.7 (C-12); 144.0 (C-13); 42.1 (C-14); 28.1 (C-15); 23.2 (C-16); 46.9 (C-17); 41.6 (C-18); 46.0 (C-19); 30.6 (C-20); 33.8 (C-21); 32.4 (C-22); 66.3 (C-23); 14.2 (C-24); 17.5 (C-25); 17.6 (C-26); 25.9 (C-27); 176.4 (C-28); 32.9 (C-29); 23.5 (C-30); sugar signals: δ 95.6 (C-1’); 74.0 (C-2’); 78.8 (C-3’); 71.0 (C-4’); 79.2 (C-5’); 62.1 (C-6’). Compound **8**: ^1^H-NMR: δ 4.25* (1H, m, H-2); 4.28* (1H, m, H-3); 5.43 (1H, brs, H-12); 2.52 (1H, d, *J* = 11.2 Hz, H-18); 3.72 (1H, d, *J* = 10.6, H-23a); 4.22* (1H, d, *J* = 10.6, H-23b); 1.07 (3H, s, H-24); 1.11 (3H, s, H-25); 1.20 (3H, s, H-26); 1.10 (3H, s, H-27); 0.92 (3H, d, *J* = 6.0 Hz, H-29); 0.88 (3H, brs, H-30); sugar signals: δ 6.27 (1H, d, *J* = 9.0 Hz, H-1’); 4.21 (1H, m, H-5’); 4.22-4.46* (5H, m H-2’, H-3’, H-4’, H-6’) *overlapping signals; ^13^C-NMR: δ 47.9 (C-1); 68.8 (C-2); 78.1 (C-3); 43.5 (C-4); 48.1 (C-5); 18.4 (C-6); 33.0 (C-7); 40.1 (C-8); 47.8 (C-9); 38.2 (C-10); 23.7 (C-11); 125.9 (C-12); 138.3 (C-13); 42.4 (C-14); 28.5 (C-15); 24.5 (C-16); 47.7 (C-17); 53.2 (C-18); 39.2 (C-19); 39.0 (C-20); 30.7 (C-21); 36.6 (C-22); 66.3 (C-23); 14.2 (C-24); 17.3 (C-25); 17.5 (C-26); 23.6 (C-27); 176.1 (C-28); 17.2 (C-29); 21.1 (C-30); sugar signals: δ 95.6 (C-1’); 73.9 (C-2’); 78.8 (C-3’); 71.1 (C-4’); 79.1 (C-5’); 62.1 (C-6’).

*Bellericoside* (**4**): colorless amorphous powder; ^1^H- and ^13^C-NMR data in accordance with the literature [22]. ^1^H-NMR: δ 4.07 (1H, m, H-2); 4.30* (1H, m, H-3); 5.42 (1H, brs, H-12); 3.18 (1H, dd, *J* = 13.1, 3.7 Hz, H-18); 4.24 (1H, d, *J* = 10.8 Hz, H-23a); 4.86 (1H, d, *J* = 10.8, H-23b); 3.97 (1H, d, *J* = 11.0 Hz, H-24a); 4.61 (1H, d, *J* = 11.0 Hz, H-24b); 1.13 (3H, s, H-25); 1.14 (3H, s, H-26); 1.16 (3H, s, H-27); 0.87 (6H, s, H-29, H-30); sugar signals: δ 6.32 (1H, d, *J* = 9.0 Hz, H-1’); 4.20 (1H, m, H-2’); 4.25-4.44* (4H, m, H-3’, H-4’, H-6’); 4.02 (1H, m, H-5’) *overlapping signals; ^13^C-NMR: δ 47.8 (C-1); 69.0 (C-2); 79.7 (C-3); 47.8 (C-4); 48.2 (C-5); 19.2 (C-6); 32.5 (C-7); 40.0 (C-8); 48.3 (C-9); 38.1 (C-10); 23.3 (C-11); 122.7 (C-12); 144.1 (C-13); 42.1 (C-14); 28.1 (C-15); 24.1 (C-16); 46.9 (C-17); 41.7 (C-18); 46.1 (C-19); 30.7 (C-20); 33.7 (C-21); 33.1 (C-22); 64.3 (C-23); 62.8 (C-24); 17.3 (C-25); 17.4 (C-26); 26.0 (C-27); 176.5 (C-28); 33.0 (C-29); 23.6 (C-30).

*Chebuloside II* (**5**): colorless amorphous powder; ^1^H- and ^13^C-NMR data in accordance with the literature [23].^1^H-NMR: 4.43 (1H, m, H-2); 4.25 (1H, d, *J* = 9.0 Hz, H-3); 5.10 (1H, brs, H-6), 3.17 (1H, dd, *J* = 13.8, 3.7, H-18); 4.04 (1H, d, *J* = 10.8 Hz, H-23a); 4.40 (1H, d, *J* = 10.8, H-23b); 1.74 (3H, s H-24); 1.78, 3H, s, H-25; 1.71 (3H, s, H-26); 1.16 (3H, s, H-27); 0.86 (3H, s, H-29); 0.85 (3H, s, H-30); sugar signals: δ 6.27 (1H, d, *J* = 9.0 Hz, H-1’); 4.20 (1H, m, H-2’); 4.28 (1H, m, H-3’); 4.30 (1H, m, H-4’); 4.00 (1H, m, H-5’); 4.40 and 4.45 (1H each, m, H-6’); ^13^C-NMR: δ 50.1 (C-1); 69.0 (C-2); 78.2 (C-3); 44.5 (C-4); 48.7 (C-5); 67.5 (C-6); 41.0 (C-7); 39.4 (C-8); 48.8 (C-9); 38.1 (C-10); 24.0 (C-11); 124.2 (C-12); 143.5 (C-13); 42.7 (C-14); 28.2 (C-15); 23.4 (C-16); 47.0 (C-17); 41.8 (C-18); 46.2 (C-19); 30.7 (C-20); 34.0 (C-21); 32.4 (C-22); 66.1 (C-23); 15.9 (C-24); 18.8 (C-25); 19.0 (C-26); 26.0 (C-27); 176.4 (C-28); 33.0 (C-29); 23.6 (C-30) .

*β-d-glucopyranosyl 2α,3β,6β-trihydroxyolean-12-en-28-oate* (**6**): colorless amorphous powder; ^1^H- and ^13^C-NMR data in accordance with the literature [24]. ^1^H-NMR: δ 4.30 (1H, m, H-2); 3.40 (1H, d, *J* = 9.0 Hz, H-3); 4.82 (1H, brs, H-6), 2.50 (2H, m, H-7); 5.50 (1H, brs, H-12); 3.19 (1H, dd, *J* = 13.5, 4.1 Hz, H-18); 5.54 (1H, brs, OH-6); 1.44 (3H, s, H-23); 1.76 (3H, s, H-24); 1.71 (3H, s, H-25); 1.68 (3H, s, H-26); 1.23 (3H, s, H-27); 0.92 (3H, s, H-29); 0.88 (3H, s, H-30); sugar signals: 6.27 (1H, d, *J* = 9.0 Hz, H-1’); 4.18-4.45 (5H, m, H-2’, H-3’, H-4’, H-6’); 4.00 (1H, m, H-5’); ^13^C-NMR: δ 50.0 (C-1); 68.7 (C-2); 84.0 (C-3); 39.5 (C-4); 57.0 (C-5); 67.5 (C-6); 41.2 (C-7); 40.6 (C-8); 48.6 (C-9); 38.3 (C-10); 23.9 (C-11); 123.2 (C-12); 143.5 (C-13); 42.7 (C-14); 28.1 (C-15); 23.4 (C-16); 46.9 (C-17); 41.7 (C-18); 46.2 (C-19); 30.6 (C-20); 34.0 (C-21); 32.4 (C-22); 29.1 (C-23); 18.5 (C-24); 18.7 (C-25); 19.1 (C-26); 26.0 (C-27); 176.3 (C-28); 33.0 (C-29); 23.5 (C-30); sugar signals: δ 95.7 (C-1’); 74.0 (C-2’); 78.6 (C-3’); 71.1 (C-4’); 79.1 (C-5’); 62.0 (C-6’).

*Bellericoside* (**4**) and *β-d-glucopyranosyl 2α,3β,23,24-tetrahydroxyurs-12-en-28-oate* (**9**) were obtained as a mixture: colorless amorphous powder. Compound (**9**): ^1^H-NMR (pyridine-*d_5_*): δ 5.45 (1H, brs, H-12); 3.18 (1H, dd, J = 13.0, 3.5 Hz, H-18); 4.05 (1H, m, H-2); 4.30* (1H, m, H-3); 4.24 (1H, d, *J* = 10.8 Hz, H-23a); 4.90 (1H, d, *J* = 10.8, H-23b); 3.95 (1H, d, *J* = 11.0 Hz, H-24a); 4.62 (1H, d, *J* = 11.0 Hz, H-24b); 1.15 (3H, s, H-25); 1.19 (3H, s, H-26); 1.10 (3H, s, H-27); 0.91 (3H, d, *J* = 6.0 Hz, H-29); 0.87 (3H, s, H-30); sugar signals: δ 6.30 (1H, d, *J* = 9.0 Hz, H-1’); 4.20 (1H, m, H-2’); 4.25-4.44* (4H, m, H-3’, H-4’, H-6’); 4.02 (1H, m, H-5’) *overlapping signals;. ^13^C-NMR (pyridine-*d_5_*): δ 47.4 (C-1), 69.0 (C-2); 79.7 (C-3); 47.8 (C-4); 48.0 (C-5); 19.2 (C-6); 33.5 (C-7); 40.2 (C-8); 48.2 (C-9); 38.0 (C-10); 23.9 (C-11); 125.9 (C-12); 138.4 (C-13); 42.4 (C-14); 28.6 (C-15); 24.6 (C-16); 47.8 (C-17); 53.2 (C-18); 39.3 (C-19); 39.1 (C-20); 30.7 (C-21); 36.7 (C-22); 64.3 (C-23); 62.9 (C-24); 17.3 (C-25); 17.4 (C-26); 23.7 (C-27); 176.2 (C-28); 17.5 (C-29); 21.2 (C-30); sugar signals: δ 95.7 (C-1’); 74.0 (C-2’); 78.8 (C-3’); 71.2 (C-4’); 79.1 (C-5’); 62.3 (C-6’).

*Betulinic acid* (**10**): colorless amorphous powder; ^1^H- and ^13^C-NMR data in accordance with those of an authentic sample and with literature [3,4,17]. ^1^H-NMR (pyridine-*d_5_*): δ 3.47 (1H, brt, *J* = 7.7 Hz, H-3); 3.55 (1H, m, H-18); 4.78 (1H, brs, H-29ª); 4.96 (1h, brs, H-29b); 1.24, (3H, s, H-23); 1.02 (3H, s, H-24); 0.83 (3H, s, H-25); 1.07*(3H, s, H-26); 1.08* (3H, s, H-27); 1.80 (3H, s, H-30) *interchangeable signals; ^13^C-NMR (pyridine-*d_5_*): δ 39.3 (C-1), 28.3 (C-2); 78.2 (C-3); 39.6 (C-4); 56.0 (C-5); 18.8 (C-6); 34.9 (C-7); 41.2 (C-8); 51.0 (C-9); 37.6 (C-10); 21.3 (C-11); 26.2 (C-12); 38.7 (C-13); 42.9 (C-14); 31.3 (C-15); 32.9 (C-16); 56.7 (C-17); 47.8 (C-18); 49.8 (C-19); 152.5 (C-20); 30.3 (C-21); 37.6 (C-22); 28.7 (C-23); 16.4 (C-24); 16.5 (C-25); 16.5 (C-26); 15.0 (C-27); 178.9 (C-28); 110.0 (C-29); 19.5 (C-30).

*Quadranoside I* (**11**): colorless amorphous powder; ^1^H- and ^13^C-NMR data in accordance with the literature [13]. ^1^H-NMR: δ 4.25 (1H, m, H-2); 3.43 (1H, d, *J* = 9.6 Hz, H-3); 4.81 (1H, brs, H-6); 5.50 (1H, d, *J* = 3.3 Hz, OH-6); 4.76 (1H, brs, H-29a); 4.90 (1H, brs, H-29b); 1.76 (6H, s, H-23, H-30); 1.45 (3H, s, H-24); 1.58 (3H, s, H-25); 1.78 (3H, s, H-26); 1.04 (3H, s, H-27); sugar signals: 6.42 (1H, d, *J* = 8.1 Hz, H-1’); 4.22 (1H, m, H-2’); 4.27 (1H, m, H-3’); 4.35 (1H, m, H-4’); 4.06 (1H, m, H-5’); 4.38-4.45 (2H, m, H-6’); ^13^C-NMR: δ 50.4 (C-1), 69.0 (C-2); 84.1 (C-3); 40.7 (C-4); 56.6 (C-5); 67.9 (C-6); 42.4 (C-7); 40.7 (C-8); 51.7 (C-9); 38.6 (C-10); 21.4 (C-11); 26.2 (C-12); 37.5 (C-13); 43.0 (C-14); 30.2 (C-15); 32.2 (C-16); 57.0 (C-17); 49.9 (C-18); 47.4 (C-19); 150.9 (C-20); 30.8 (C-21); 36.8 (C-22); 28.8 (C-23); 19.0 (C-24); 19.3 (C-25); 17.0 (C-26); 15.2 (C-27); 174.9 (C-28); 110.0 (C-29); 19.4 (C-30); sugar signals: δ 95.4 (C-1’); 74.3 (C-2’); 78.8 (C-3’); 70.9 (C-4’); 79.3 (C-5’); 62.2 (C-6’).

### Antifungal assay

The following strains from the American Type Culture Collection (ATCC), Rockville, MD, USA were used for the antifungal evaluations: *Candida albicans* (ATCC 90028), *C. krusei* (ATCC 6258) and *Cryptococcus neoformans* (ATCC 32045). The antifungal activities were determined using microbroth dilution assays in 96-well microplates, in duplicate, following the guidelines of the Clinical and Laboratory Standards Institute (CLSI) [36]. The lowest concentration of compound at which no fungal growth was observed after incubation was recorded as the minimum inhibitory concentration (MIC). Amphotericin B was used as the reference antimycotic control.
